# Supplementary material for: Investigating the influence of physiologically relevant hydrostatic pressure on CHO cell batch culture
Source: Sci Rep. 2021 Jan 8;11:162. doi: 10.1038/s41598-020-80576-8 (PMC7794228; doi:10.1038/s41598-020-80576-8)
Supplement: Supplementary file 1 — Supplementary Information 1. [file 41598_2020_80576_MOESM1_ESM.zip › S1/IgG/Feb14 0 30 60 90/60mmHg Feb8.pdf]

Data File C:\Users\P...emStation\1\Data\TK_CHO_IgG 2019-02-14 14-17-55\022-12-ML 60 Feb8.D
Sample Name: ML 60 Feb8

===

Acq. Operator : SYSTEM   Seq. Line : 22
Sample Operator : SYSTEM
Acq. Instrument : HPLC-0039   Location : 12
Injection Date : 2/14/2019 3:49:22 PM   Inj : 1
                                          Inj Volume : 100.000 µl
Method : C:\Users\Public\Documents\ChemStation\1\Data\TK_CHO_IgG 2019-02-14 14-17-55
\CHO_IgG_LC.M (Sequence Method)
Last changed : 2/8/2019 3:17:23 PM by SYSTEM

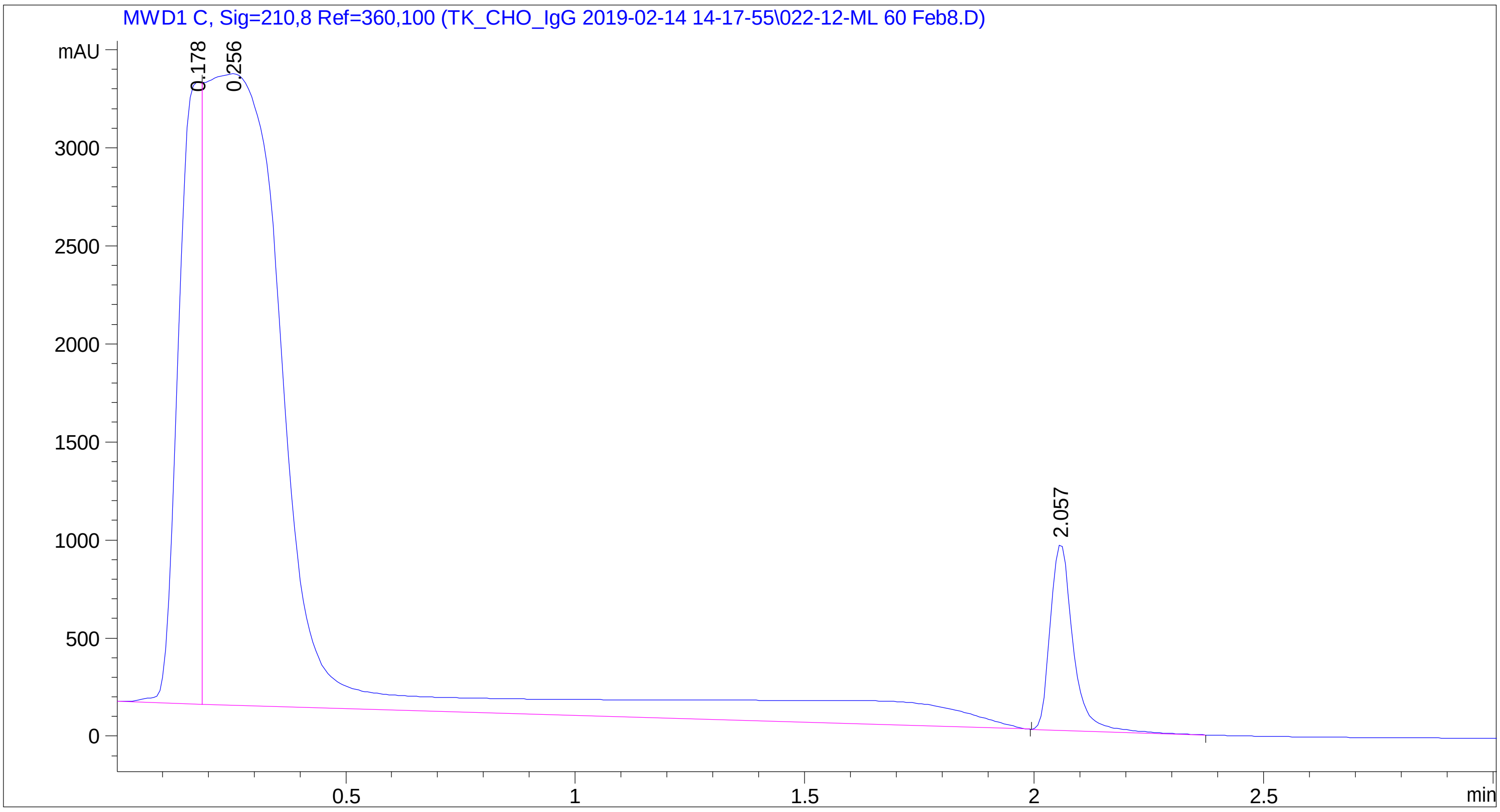

===

Area Percent Report

===

Sorted By : Signal
Multiplier : 1.0000
Dilution : 1.0000
Use Multiplier & Dilution Factor with ISTDs

Signal 1: MWD1 C, Sig=210,8 Ref=360,100

Peak #	RetTime [min]	Type	Width [min]	Area [mAU*s]	Height [mAU]	Area %
1	0.178	BV	0.0549	1.06728e4	3170.90381	18.6195
2	0.256	VB	0.1911	4.33517e4	3218.82202	75.6302
3	2.057	BBA	0.0544	3296.10034	951.55304	5.7503

Totals : 5.73207e4 7341.27887

===

*** End of Report ***
